# Supplementary material for: The Novel Z-Scheme Ternary-Component Ag/AgI/α-MoO3 Catalyst with Excellent Visible-Light Photocatalytic Oxidative Desulfurization Performance for Model Fuel
Source: Nanomaterials (Basel). 2019 Jul 23;9(7):1054. doi: 10.3390/nano9071054 (PMC6669729; doi:10.3390/nano9071054)
Supplement: Supplementary file 1 [file nanomaterials-09-01054-s001.pdf]

Submitted to ***Nanomaterials***

Electronic Supplementary Information for

The Novel Z-Scheme Ternary-Component Ag/AgI/α-MoO_3 Catalyst with Excellent Visible-Light Photocatalytic Oxidation Desulfurization Performance for Model Fuel

Yanzhong Zhen[*1], Jie Wang [1], Feng Fu[*1], Wenhao Fu[1] and Yucang Liang[*2],

[1] Shaanxi Key Laboratory of Chemical Reaction Engineering, School of Chemistry & Chemical Engineering, Yan'an University, Yan'an 716000, Shaanxi, China

[2] Institut für Anorganische Chemie, Eberhard Karls Universität Tübingen, Auf der Morgenstelle 18, 72076 Tübingen, Germany

* To whom correspondence should be addressed.

E-mail: zyz943@163.com (Associate Prof. Dr. Y. Z.)
yadxfufeng@126.com (Prof. Dr. F. F.)
yucang.liang@uni-tuebingen.de (Dr. Y. L.)

Captions and Figures:

Fig. S1. XPS survey spectrum of $Ag/AgI/\alpha\text{-}MoO_3$ (AAM-35).

Fig. S2. Comparison of XRD patterns of white precipitates in $Ba(NO_3)_2$ solution and AAM-35.

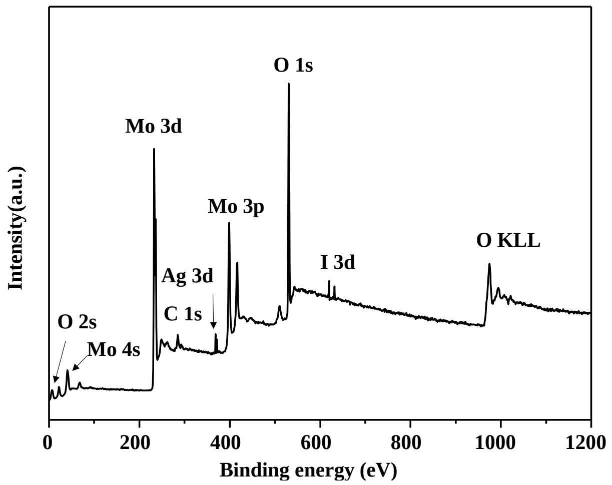

Fig. S1. XPS survey spectrum of Ag/AgI/α-MoO_3 (AAM-35)

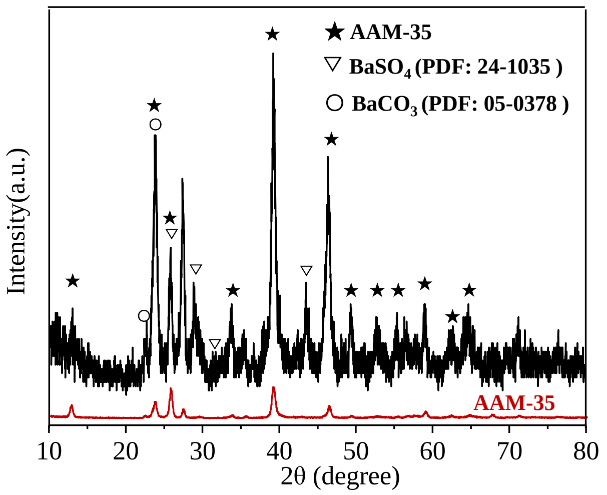

Fig. S2. Comparison of XRD pattern of white precipitates in $Ba(NO_3)_2$ solution and pure AAM-35.
